# Structure and Deformation Behavior of Ti-SiC Composites Made by Mechanical Alloying and Spark Plasma Sintering

**DOI:** 10.3390/ma12081276

**Published:** 2019-04-18

**Authors:** Dariusz Garbiec, Volf Leshchynsky, Alberto Colella, Paolo Matteazzi, Piotr Siwak

**Affiliations:** 1 Metal Forming Institute, 14 Jana Pawla II St., 61-139 Poznan, Poland; leshchynsky@inop.poznan.pl; 2 MBN Nanomaterialia, 42 Via G. Bortolan, 31050 Vascon Di Carbonera, Italy; alberto.colella@matres.org (A.C.); matteazzi@mbn.it (P.M.); 3 Poznan University of Technology, 5 Marii Sklodowskiej-Curie Square, 60-965 Poznan, Poland; piotr.siwak@put.poznan.pl

**Keywords:** MAX phase, Ti_3_SiC_2_, composite, high energy ball milling, spark plasma sintering, structure, mechanical properties, deformation behavior

## Abstract

Combining high energy ball milling and spark plasma sintering is one of the most promising technologies in materials science. The mechanical alloying process enables the production of nanostructured composite powders that can be successfully spark plasma sintered in a very short time, while preserving the nanostructure and enhancing the mechanical properties of the composite. Composites with MAX phases are among the most promising materials. In this study, Ti/SiC composite powder was produced by high energy ball milling and then consolidated by spark plasma sintering. During both processes, Ti_3_SiC_2_, TiC and Ti_5_Si_3_ phases were formed. Scanning electron microscopy, energy-dispersive X-ray spectroscopy and X-ray diffraction study showed that the phase composition of the spark plasma sintered composites consists mainly of Ti_3_SiC_2_ and a mixture of TiC and Ti_5_Si_3_ phases which have a different indentation size effect. The influence of the sintering temperature on the Ti-SiC composite structure and properties is defined. The effect of the Ti_3_SiC_2_ MAX phase grain growth was found at a sintering temperature of 1400–1450 °C. The indentation size effect at the nanoscale for Ti_3_SiC_2_, TiC+Ti_5_Si_3_ and SiC-Ti phases is analyzed on the basis of the strain gradient plasticity theory and the equation constants were defined.

## 1. Introduction

During the last two decades, nanocrystalline materials with grain sizes below 100 nm prepared using various methods have been widely studied due to their enhanced properties when compared with coarse-grained polycrystalline materials. Mechanical milling/alloying (MM/MA) is an effective method to prepare nanostructured materials such as pure element nanocrystalline metals, supersaturated solid solutions, intermetallic compounds, dispersion strengthened alloys and amorphous alloys. Mechanochemical synthesis—chemical reactions induced by high energy ball milling (HEBM)—is one of the promising routes for the synthesis of different materials and composites such as M_n+1_AX_n_ ternary layered compounds, where n=1, 2 or 3, M is an early transition metal, A is an A-group element (mostly groups 13 and 14), and X is C or N. Mechanochemical reactions may be divided into by two categories [1], specifically: (i) those which occur during the mechanical activation process, and (ii) those which occur during subsequent thermal treatment [2]. Indeed, the material grain size influences the mechanochemical reactions of both categories. Applying the spark plasma sintering (SPS) route has proved [3] to be extremely important to retaining the nanostructure of composites and to achieve their unique properties due to the formation of various phases during the sintering process [4].

Despite the fact that Ti and its alloys are widely used in many areas of engineering, the mechanical properties of these materials could be insufficient in some structural applications, especially at elevated temperatures [5]. One of the opportunities to improve the mechanical properties of Ti and its alloys could be to reinforce them with ceramics [6]. The most frequently used ceramic reinforcements are TiC [7] and SiC [6]. SiC ceramic reinforcement may be in the form of fibers, which provides high specific strength and stiffness, but only in the direction of the fibers [6,8]. The main limitations of the fiber-type reinforcement of Ti-based composites are the high cost, non-formability and difficultly of machining [9]. A better choice is to use SiC particles. The SiC ceramic can react with Ti during powder metallurgy processing routes. One of the results of chemical reactions between Ti and SiC during sintering is the formation of MAX phases.

MAX phases have attracted much attention because of their unique combination of both metal- and ceramic-like properties [10], such as high fracture toughness, high Young’s moduli, high thermal and electrical conductivities, easy machinability, excellent thermal shock resistance, high damage tolerance, and microscale ductility [11]. In particular, the fact that MAX phases exhibit properties, which are typical both of metals and ceramics became known during the last two decades thanks to the studies of Prof. Barsoum et al. (Drexel University, USA) [12]. This group of researchers found that MAX phases are natural nanolaminates and have high electrical and heat conductivity, and lower coefficients of friction as compared to known hard materials, high rigidity in combination with a low density and high fracture toughness [13]. To synthesize bulk MAX phases, various methods have been used, e.g., both cold compacting and pressureless sintering [14], hot pressing (HP) [15], hot isostatic pressing (HIP) [16] and the field assisted sintering technology (FAST) [17]. In some cases, the self-propagating high-temperature synthesis (SHS) reaction also occurred [18]. However, the structure of MAX phase-based composites usually consists of various phases, and the influence of these phases on the mechanical properties of composites has not been studied in detail.

The objective of this work is to analyze the Ti_3_SiC_2_ MAX phase-based composite deformation behavior based on Vickers nanohardness and microhardness measurements of SPSed composites. The sintering process of an HEBMed Ti/SiC nanostructured composite powder, the influence of the sintering temperature on the Ti_3_SiC_2_ MAX phase-based composite structure and the mechanical properties (nanohardness of various phases, microhardness and fracture toughness) are analyzed.

## 2. Materials and Methods

HEBMed Ti/SiC (50 vol%) nanostructured composite powder was developed and supplied by MBN Nanomaterialia and then SPSed in vacuum using an HP D 25-3 furnace (FCT Systeme, Rauenstein, Germany). To avoid particle oxidation, the powder was transferred from the manufacturing facility in a specially sealed parcel under vacuum, and the parcel was opened directly before filling the graphite die. The sintering temperatures of 1350, 1400 and 1450 °C were applied at a heating rate of 200 °C/min. The holding time was 10 min. Afterwards, the SPSed compacts were cooled to ambient temperature. The compacting pressure of 50 MPa was applied throughout all the sintering stages. Discs of a diameter of 20 mm and thickness of 5 mm were produced.

The agglomerate size distribution was measured by laser diffraction using a Mastersizer 2000 analyzer (Malvern Panalytical, Malvern, UK). X-ray structural studies of the powder and the SPSed compacts were conducted using a Kristalloflex 4 diffractometer (Siemens, Berlin, Germany) using MoK_α_ radiation with 2Θ 15–50°. Microscopic observations and elemental microanalysis were performed using two scanning electron microscopes: an Inspect S (FEI, Hillsboro, TX, USA) and a MIRA3 (TESCAN, Brno, Czech Republic) equipped with an energy-dispersive X-ray spectroscope (EDS) (EDAX, Mahwah, NJ, USA). The effective density measurements were made by the Archimedes method. The Vickers hardness measurements were carried out using an FV-800 hardness tester (Future-Tech, Kawasaki, Japan). The applied loads were 0.4903, 4.903 and 19.61 N and the holding time was 15 s. The fracture toughness was calculated based on the crack length measured from the corner of the indentation made by a Vickers indenter using Equation (1) [19]:

(1) $$K_{1c}=0.15\sqrt{\frac{HV_{30}}{\sum l}}$$

where *HV*_30_ is the Vickers hardness measured under the load of 294.2 N and *l* is the total length of cracks initiated from the corners of the indenter. The Vickers indentation nanohardness measurements were performed using a Picodentor HM500 nanoindenter (Fisher, Windsor, SC, USA). The applied load was 0.05 N and the holding time was 5 s.

It is well known today that materials exhibit properties on the sub-micro and nanoscale, which are entirely different from those exhibited on the macroscale. One of the main effects exhibiting this difference is the increase in hardness with a decrease in indentation load or depth, known as the indentation size effect (ISE). Various nano- and microstructural features, such as lattice defects, grain boundaries, interfacial effects, phase structure and composition, etc., are responsible for such behavior of a material. That is why the ISE examination of Ti_3_SiC_2_ MAX phase-based composites is extremely important to understand the scientific issues involved in the genesis of size effects similar to work [20].

**Figure 1:**
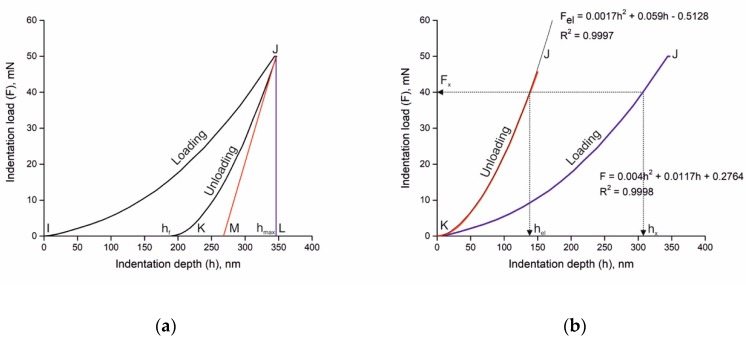
Indentation curve schematics: indentation curve of Ti_3_SiC_2_ MAX phase of specimen SPSed at 1350 °C (**a**) and approximation and calculation of indentation parameters (**b**).

The nanoindentation process is described in Figure 1 by the dependence of load *F* applied to the nanoindenter tip to depth *h* of tip penetration in a given sample (Figure 1a). In the present work, a Vickers tip was used. Loading started at point *I* and continued along the loading arrow up to the point *J* where *F* became *F_max_*. The maximum depth of penetration is shown as *h_max_*. It was identified by the perpendicular *JL* drawn on the depth *h* axis. The unloading curve defined the path of unloading up to *K* where the load was reduced to zero while the final depth was elastically recovered from *h_max_* at *L* to *h_f_* at *K* (Figure 1a). Calculation of plastic deformation depth *h_pl_* = *h_x_* − *h_el_* was made on the basis of determining square root *h_el_* of the equation describing the unloading curve (Figure 1b).

## 3. Results and Discussion

### 3.1. Reactions of Sintering Process

**Figure 2:**
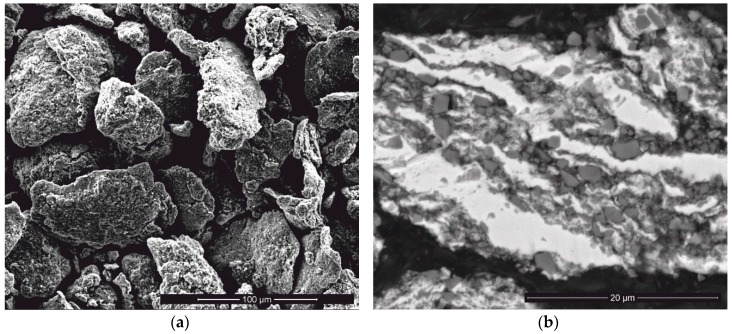
Morphology (**a**) and internal/cross cut structure (**b**) of Ti/SiC HEBMed powder agglomerates.

Figure 2 presents the morphology and structure of the Ti/SiC HEBM powder. This powder consists of agglomerates with both, flaky and granular shaped particles (Figure 2a). The agglomerate size distribution of the HEBMed powder is presented in Table 1. The average size of the agglomerates is 42.2 μm, but some of the agglomerate sizes are close to 100 μm. The agglomerate structure analysis reveals that the SiC particles are randomly distributed in the Ti matrix and their sizes vary in a wide range (Figure 2b). Areas with a high content of SiC particles are adjacent to pure Ti. These Ti/SiC HEBMed powder feedstock structure features influence the further Ti-SiC reaction regime during sintering.

**Figure 3:**
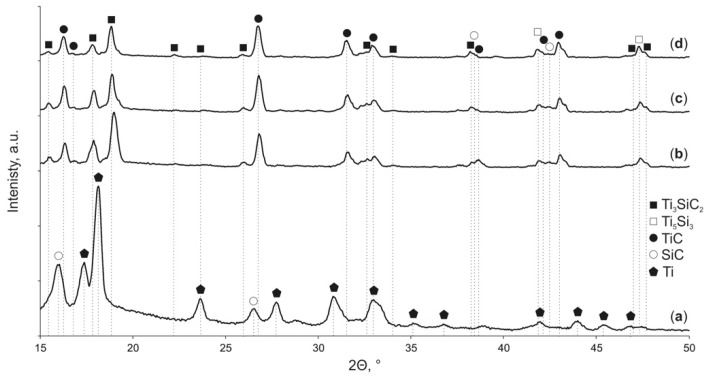
X-ray diffraction spectra of: Ti/SiC HEBMed powder (**a**); composite SPSed at 1350 °C (**b**); composite SPSed at 1400 °C (**c**) and composite SPSed at 1450 °C (**d**).

The X-ray spectra of the HEBM Ti/SiC powder (Figure 3) demonstrate that the powder feedstock consists of only Ti and SiC. Therefore, the chosen HEBM regime of the Ti/SiC powder did not lead to Ti-SiC reactions, and this powder feedstock was used for further SPS.

**Figure 4:**
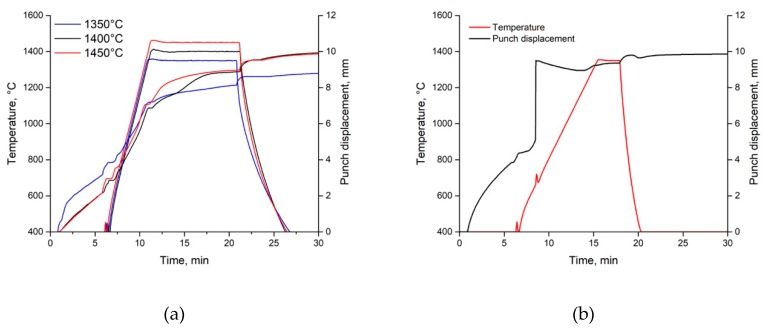
Punch displacement and temperature change during SPS of Ti/SiC HEBM powder at 1350, 1400 and 1450 °C and 10 min (**a**) and at 1350 °C and 2.5 min (**b**).

The densification behavior of the Ti-SiC composite during SPS was estimated based on analysis of the punch displacement and temperature dependences on the sintering time shown in Figure 4. The following process stages are seen: (i) air removal, (ii) pressing, (iii) heating, and (iv) holding at the sintering temperatures of 1350, 1400 and 1450 °C, and, subsequently, (v) cooling (Figure 4a). The punch displacement diagram for sintering at 1350 °C and 2.5 min (Figure 4b) demonstrates an SHS-type reaction, which is characterized by a considerable increase in the densification rate (punch displacement rate) and temperature peak at ~700 °C and diffusion reactions during holding at the temperature of 1300–1450 °C.

**Figure 5:**
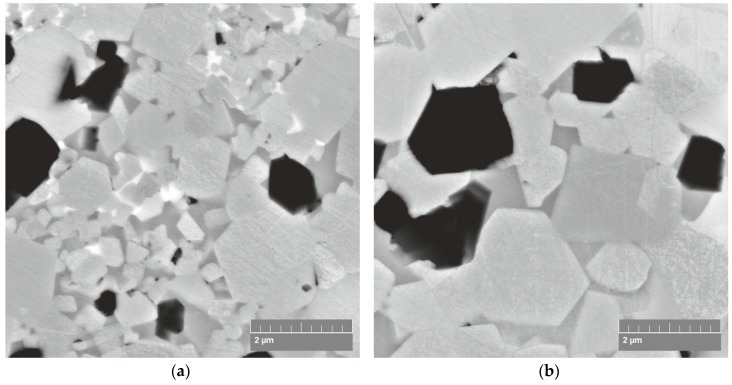

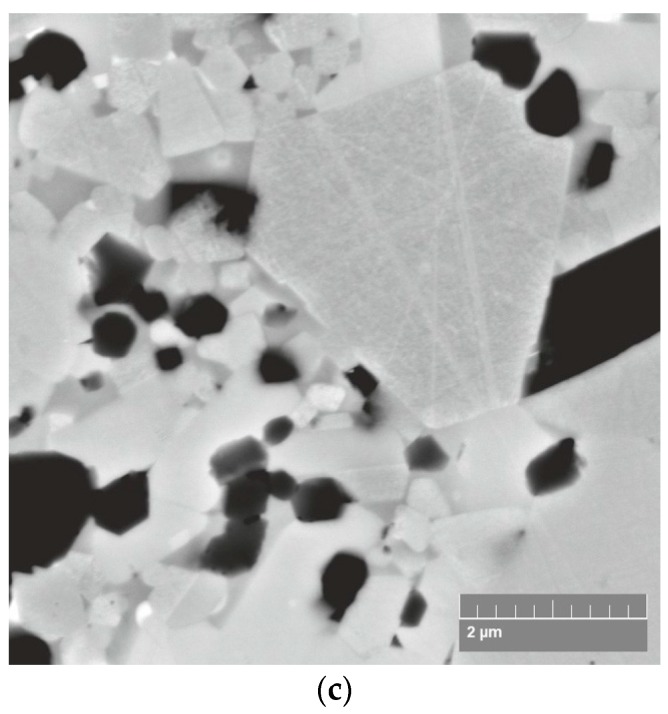
SEM micrographs of composites SPSed at: 1350 °C (**a**); 1400 °C (**b**) and 1450 °C (**c**).

**Figure 6:**
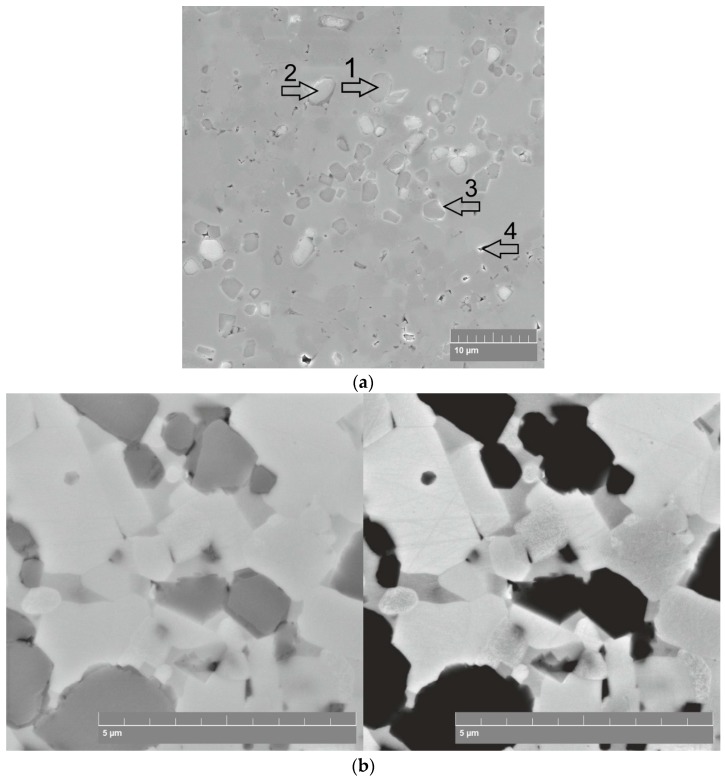
SEM micrographs of composite SPSed at 1400 °C: in-beam secondary electrons with magnification 5kx, arrow #1—dark phase, arrow #2—grey phase, arrow #3—bright phase and arrow #4—pore area (**a**) and comparison of in-beam secondary electrons and backscattered electrons micrographs (**b**).

The X-ray diffraction analysis results (Figure 3) reveal the formation of a three-phase structure. It was found that the phase composition of the SPSed composites consists of dominant of Ti_3_SiC_2_ and TiC and Ti_5_Si_3_ phases. The results of the structure examination of the SPSed composites are shown in Figure 5 and Figure 6. The data reveal the following specific features of the sintering process: (i) the dissolution of small SiC particles formed due to HEBM and the formation of new crystals during sintering, (ii) the presence of relatively large SiC-based particles that demonstrates their stability at the chosen sintering temperatures, (iii) the complexity of the chemical composition of the sintered phases due to the multiple diffusion of Ti, Si and C atoms, and (iv) grain growth of the MAX-based phase at high sintering temperatures.

The reactions between a Ti-based matrix and SiC particles were determined in works [21,22,23]. Gottselig et al. [21] studied the reaction behavior of Ti sputtered on SiC. It was found that mainly the ternary Ti_3_SiC_2_ MAX phase (>90%) is formed in the sintering temperature range between 1250 and 1500 °C. In experiments joining SiC with Ti layers at 1450 °C and at compacting pressures between 5 and 30 MPa, the formation of the ternary MAX phase leads to a high joining strength. A high sintering temperature results in fast diffusion of the Si and C atoms from the SiC particles into the Ti matrix formed due to severe plastic deformation of the Ti particles during the HEBM process. The milled SiC particles of a small size have an SHS reaction with Ti similar to those described in [24]. This SPS structure formation process is similar to the SPS joining described by Zhao et al. [23]. The diffusion layer at the Ti/SiC interface is shown [23] to be stratified at 1400 °C. This layer was enriched with Ti and C, while depleted in Si. These results suggested the formation of a TiC layer at the interface via interdiffusion between the interlayer and the SiC particles.

The EDS analysis results shown in Table 2 demonstrate similar diffusion processes which results in reactions described in detail by Gotman et al. [22]. The main possible chemical reactions during the sintering process are shown below [23]:

(i) SiC particle surface reactions and SiC dissolution:
  8Ti + 3SiC → 3TiC + Ti_5_Si_3_;
  10Ti + 4SiC → Ti_5_Si_3_ + 2TiC + Ti_3_SiC_2_;
  2Ti + SiC + TiC → Ti_3_SiC_2_;
(ii) Reactions of Si and C atoms diffusing into Ti matrix:
  5Ti +3Si → Ti_5_Si_3_;
  Ti + C → TiC.

Gotman et al. [22] clearly showed that all the above-listed reactions are thermodynamically favored, which means that the interaction between SiC and the Ti matrix can start with the formation of both TiC and Ti_5_Si_3_. The thermodynamic calculations conducted in work [22] show that the reaction layer at the surface of the SiC particles can contain both TiC and Ti_5_Si_3_. The micrographs in Figure 5 and Figure 6 shows the bright contrast of these areas (see, for example, arrow #3 in Figure 6a) which reveals the presence of TiC and Ti_5_Si_3_ phases and the formation of a diffusion barrier layer. However, it can be seen that this barrier layer is not continuous. It is interesting to note that Ti diffusion into SiC does occur and results in obtaining a gray contrast of some SiC particles (see, for example, arrow #2 in Figure 6a), despite the presence of a TiC-Ti_5_Si_3_ barrier layer. Hence, some large dark SiC particles become gray due to a change in their chemical composition (an increase in Ti content, see Table 2). It is known that some errors occurred when determining the Ti and C content in former SiC particles by EDS. For this reason, additional EDS analysis was conducted on the fractured surfaces and showed similar results. The structure analysis results reveal that the large SiC particles cannot be completely decomposed during reaction sintering. Therefore, only particles of a size below the critical may be dissolved. Thus, the HEBM regime needs to be chosen to avoid the presence of large particles. 

**Figure 7:**
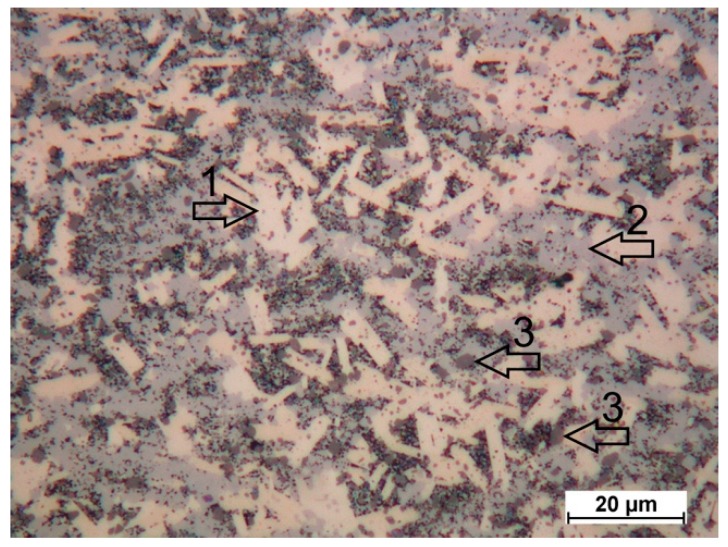
LM micrograph of composite SPSed at 1350 °C.

The main Ti_3_SiC_2_ MAX phase is believed to be formed due to the diffusion of Si and C atoms during the dissolution of small SiC particles. However, this process is not uniform, because of the extreme difference of SiC particle content in the various fields of the HEBMed powder particles (Figure 1b). Areas of small crystals are seen in the SEM micrograph of the composite SPSed at 1400 °C (Figure 6a). Some of them have a bright contrast, which indicates TiC and Ti_5_Si_3_. The same phases are seen in the light microscope micrograph of the composite SPSed at 1350 °C (Figure 7) etched with an HF-HNO_3_-H_2_O solution with the ratio HF:HNO_3_:H_2_O–1:1:5: Ti_3_SiC_2_ MAX phase—platelet bright crystals (arrow #1), areas of small crystals with the presence of TiC and Ti_5_Si_3_ (arrow #2), and former SiC particles (arrow #3).

The structure of the composites SPSed at the temperature ranging between 1350 and 1450 °C presented in Figure 5 demonstrates the effect of the sintering temperature on the grain size of the Ti_3_SiC_2_ MAX phase. The Ti_3_SiC_2_ MAX phase recrystallization effect is seen in the composites SPSed at 1400 and 1450 °C. Comparison of the SEM in-beam secondary electrons and backscattered electrons micrographs (Figure 6b) allows the ratio between Si, C and Ti to be roughly evaluated and the conclusion to be drawn that both the structure formation of the SiC particles and the Ti matrix are governed by the diffusion of Si, C into Ti and Ti into the SiC particles. Therefore, the mechanical properties of the composite are controlled by these factors.

### 3.2. Mechanical Properties of Composites

The results of the effective density, hardness and fracture toughness measurements of composites SPSed at 1350, 1400 and 1450 °C are summarized in Table 3. It can be clearly seen that increasing the sintering temperature affected an increase in the density from 4.33 to 4.42 g/cm^3^. The density of the SPSed composites varied only in the range of 0.09 g/cm^3^. For this reason, the influence of porosity on the diffusion rates of the synthesis reactions was not taken into account. The hardness also increased with raising the sintering temperature. The specimens SPSed at 1400 and 1450 °C exhibit an increase in both the Vickers hardness and fracture toughness calculated from the indentation tests. The fracture toughness of the composites reached the maximum for the specimen SPSed at 1450 °C (K_1c_ = 6.06 MPa⋅m^1/2^), whereas the fracture toughness of pure TiC is about 3.70 MPa⋅m^1/2^ [25] and the maximal fracture toughness of the Ti_3_SiC_2_ MAX phase is about 11.50 MPa⋅m^1/2^ [10,12].

**Figure 8:**
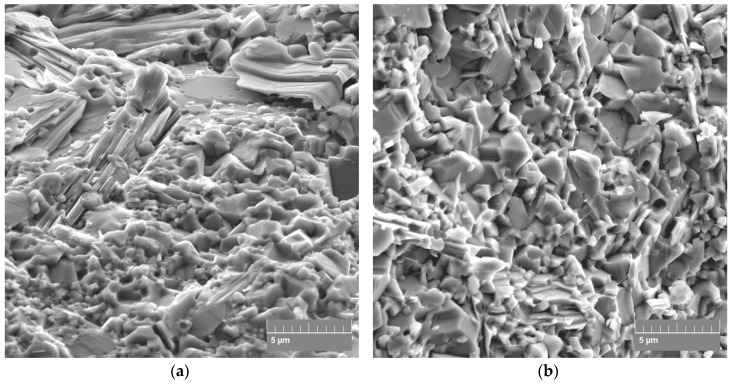

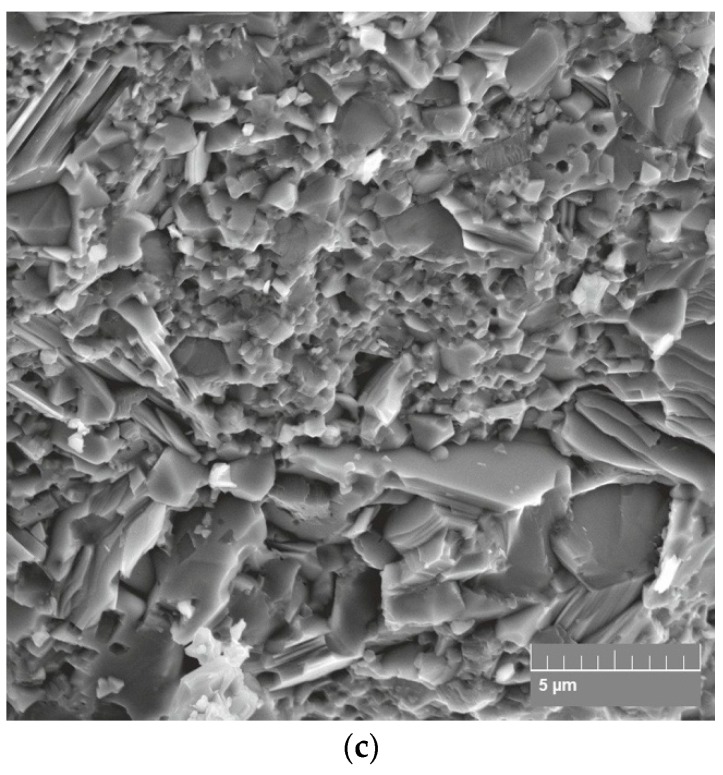
SEM micrographs of fracture surface topography of composites SPSed at: 1350 °C (**a**); 1400 °C (**b**) and 1450 °C (**c**).

The increase in fracture toughness with the sintering temperature may be attributed to deflection of the cracks due to an increase in the Ti_3_SiC_2_ MAX phase content and a possible decrease in the Ti_5_Si_3_ brittle phase concentration (Figure 8).

It is well known that the physical, structural, mechanical and other properties change drastically on the nanoscale, thereby attracting attention to the size effects in various materials, especially ceramics. Therefore, it is extremely important to clarify the basic effects of size effects in materials, particularly ceramics, which are characteristically brittle in nature. Hence, examining the ISE of nanostructured ceramic-based composites is very important from the viewpoint of synthesizing new materials. Examining the nanomechanical behavior of composite phases will allow further understanding of their deformation and fracture mechanisms and the real area of its application to be determined. Thus, the major objectives of the nanohardness analysis of the composites is: (i) to study the nanoindentation response of SPSed composites sintered at various temperatures, and (ii) to examine the strain gradient plasticity (SGP) model to explain the ISE of various phases of sintered composites. Therefore, nanohardness measurements of the three phases were performed.

**Figure 9:**
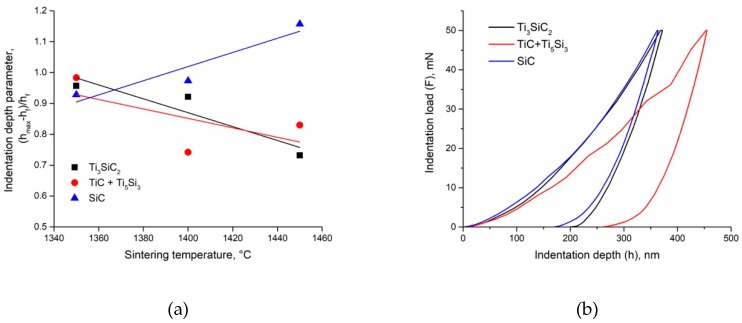
Indentation parameter (*h_max_* − *h_f_*)/*h_f_* dependence on sintering temperature (**a**) and load-penetration depth curves of phases of composite SPSed at 1400 °C (**b**).

The variation of the nanohardness of the SPSed composites as a function of load and depth is shown in Figure 1, as an example, and in Figure 9b for composites SPSed at 1400 °C. It is important to note that the phases of the SPSed composites exhibit the presence of a very strong ISE which has not yet been studied in detail. The validity of the SGP model of the ISE explanation and characterization for the SPSed composites may be evaluated by comparison of the indentation and SGP equation parameters. The data presented in Figure 9a show that in the SPSed composites the ratio of elastic to plastic depths (*h_max_* − *h_f_*)/*h_f_* increases for the SiC particles and decreases for the Ti_3_SiC_2_ MAX phase and the TiC+Ti_5_Si_3_ phase. Clearly, the lower the (*h_max_* − *h_f_*)/*h_f_* ratio is, the higher the final depth *h_f_* of penetration (Figure 9a), which indicates higher plastic strain. The data presented in Figure 9a demonstrate a similar deformation ability of the Ti_3_SiC_2_ MAX phase and TiC+Ti_5_Si_3_ phase. However, the deformation mechanisms of these phases are not clear, and the load-indentation depth diagrams of the TiC+Ti_5_Si_3_ phase contains the areas of crack generation (Figure 9b). The ISE mechanisms were analyzed by Maiti et al. [20], and the validity of various of phenomenological behaviors of nano-mechanical models (elastic recovery, proportional specimen resistance and Nix and Gao) to the experimental data of a ZrO_2_ ceramic was determined. However, only the Nix and Gao model has a proper physical background—the SGP theory developed by Fleck et al. [26] and Nix and Gao [27]. It allows some physical-based parameters of composite plastic deformation to be estimated. Their estimation on the basis of the nanohardness measurement of the Ti_3_SiC_2_ MAX phase, TiC+Ti_5_Si_3_ and SiC phases will allow the real deformation mechanisms to be determined.

In general, in SGP theory, it is assumed [27] that the indentation is accommodated by circular loops of geometrically necessary dislocations with Burgers vectors normal to the plane of the surface. As the indenter is forced into the surface of a single crystal, a certain density of geometrically necessary (GND) dislocations *ρ_G_* is required to account for the permanent shape change at the surface. Other dislocations, called statistically stored dislocations by Ashby [28], would also be created and they would contribute to the deformation resistance. The geometry of indentation contact is characterized by the following parameters: (i) the angle between the surface of the conical or pyramidal indenter and the plane of the surface *Θ*, (ii) the contact radius *a*, and (iii) the depth of indentation *h*.

To estimate the shear strength, the authors of [27] use the Taylor relation:

(2) $$\tau =\alpha \mu b\sqrt{\rho_{T}}=\alpha \mu b\sqrt{\rho_{G}+\rho_{S}}$$

where *ρ_T_* is the total dislocation density in the indentation, *ρ_S_* is the density of statistically stored dislocations, *µ* is the shear modulus, *b* is the Burgers vector and *α* is a constant (0.5). *ρ_S_* is not expected to depend on the depth of indentation. 

Taking into account the fact that a Tabor’s [29] factor of 3 can be used to convert the equivalent flow stress to hardness, the von Mises yield criterion may be presented as:

(3) $$\sigma =\sqrt{3}\tau ;H=3\sigma ⊳H=3\sqrt{3}\alpha \mu b\sqrt{\left(\rho_{G}+\rho_{S}\right)}$$

and the hardness equation can be shown as:

(4) $$\frac{H}{H_{0}}=\sqrt{1+\left(\frac{h^{*}}{h}\right)}⊳{\left(\frac{H}{H_{0}}\right)}^{2}=h^{*}\left(\frac{1}{h}\right)+1$$

where *H*_0_ is the hardness that would arise from the statistically stored dislocations alone, in the absence of any geometrically necessary dislocations, and:

(5) $$h^{*}=\frac{81}{2}b\alpha^{2}tg^{2}\theta {\left(\frac{\mu }{H_{0}}\right)}^{2}$$

where h* is a length that characterizes the depth dependence of the hardness. It must be noted that h* is not a constant for a given material or indenter geometry. It depends on the statistically stored dislocation density through *H*_0_.

**Figure 10:**
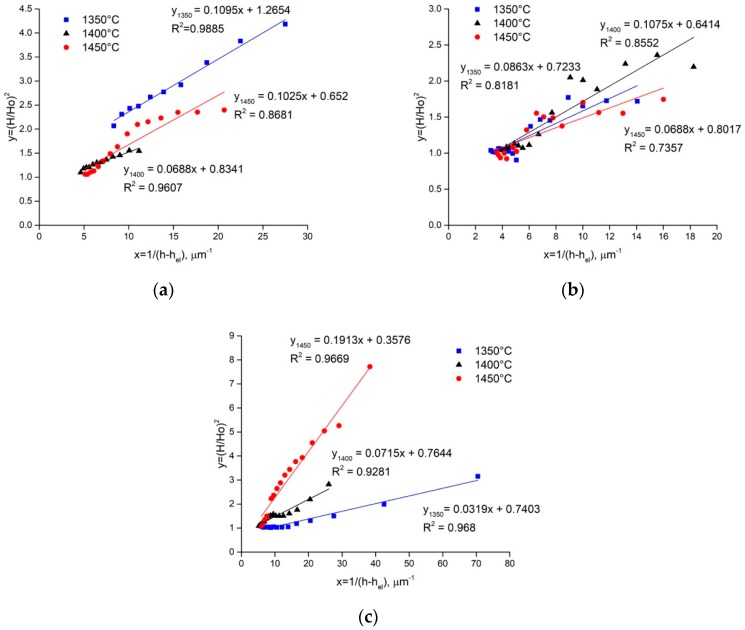
Application of Nix and Gao model to nanohardness experimental data of: Ti_3_SiC_2_ (**a**); TiC+Ti_5_Si_3_ (**b**) and SiC (**c**) phases of composites SPSed at 1350, 1400 and 1450 °C.

The validity of Nix and Gao model to the nanohardness experimental data is defined by calculating the linear approximation (*H*/*H*_0_)^2^ = *f*(*1*/(*h* − *h_f_*)) of the nanohardness measurement results (Figure 10). The results demonstrate that only the Ti_3_SiC_2_ MAX phase exhibits dependence (*H*/*H*_0_)=*f*(*1*/(*h* − *h_f_*)) close to the Nix and Gao equation [27] (Equation (4), the imulation veracity is R^2^ = 0.8680−0.9885). The fitting of the experimentally measured F-h data of the TiC+Ti_5_Si_3_ phase gives Nix and Gao parameters with a goodness of fit (R^2^) in the range of 0.7357–0.8552 (Figure 10). Slightly better results are for the SiC-based particles—R^2^ = 0.9281−0.9680. This means that the calculated strain gradient parameters of only the Ti_3_SiC_2_ MAX phase reflect the real dislocation mechanisms of deformation.

The ISE is observed in both the nanohardness and microhardness of various metals [30]. While the explanations of SGP theory shown above seem to be valid for nanoindentation [27], the mechanism of ISE for the microindentation of SPSed composites needs to be corrected taking into account the specific structure features. Some experimental results of determining the nanohardness and microhardness ISE are shown in Table 4.

The values of true hardness *H*_0_ and characteristic depth h* are shown in Table 4 for all the examined phases. The *H*_0_ values show that the true hardness parameter of all the phases is higher than that of the ZrO_2_ ceramic presented in paper by Maiti et al. [20], because it is calculated for indentation depth *h_x_* = *h* − *h_el_*. The characteristic depth h* of the Ti_3_SiC_2_ MAX phase varies in the range of h* = 0.07 − 0.11, which is in the range of h* defined for different metals–Ni, Ag, Au and Cu [31]. Parameter h* defined for other phases of the SPSed composite varies in a wide range 0.03–0.19 which indicates the weak effectiveness of the Nix and Gao deformation model for TiC+Ti_5_Si_3_ and SiC phases.

**Figure 11:**
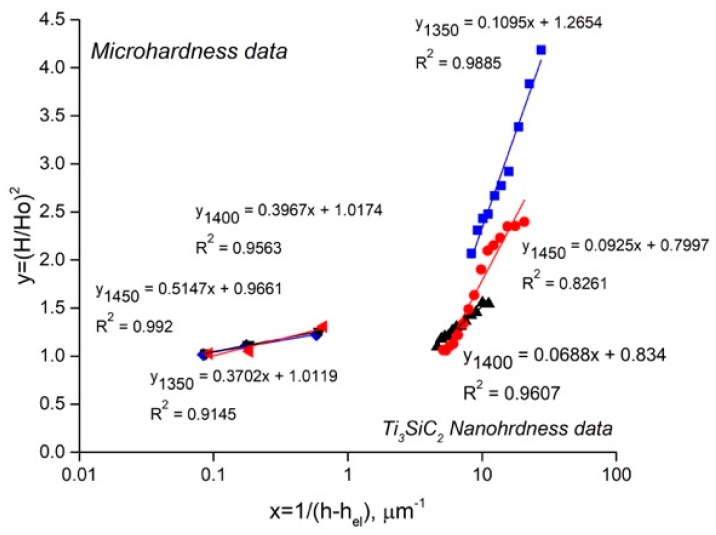
Application of the Nix and Gao model to composites and Ti_3_SiC_2_ phase of composites SPSed at 1350, 1400 and 1450 °C.

The comparison between the microhardness and nanohardness data fitting to the Nix and Gao model is shown in Figure 11. The results indicate the high values of *H*_0_ and h* for the microindentation cases, which are the result of the low dislocation density and difficulties to generate and move dislocations in the crystals. We observe regular mechanical behavior of the composite during indentation, and only a small effect of the sintering temperature. Therefore, the ISE of microhardness is not sensitive to the composite structure and is not controlled by dislocation-based mechanisms. 

**Figure 12:**
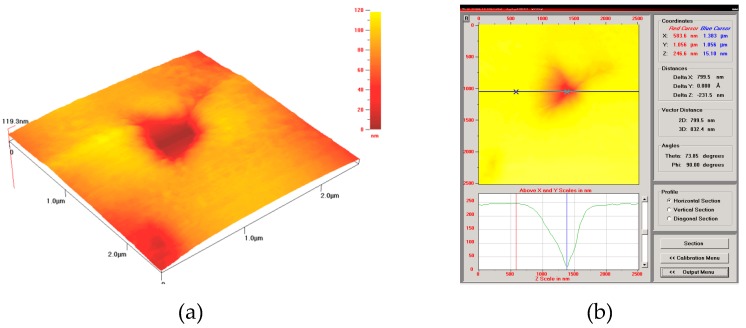
Indents (**a**) and material pile-up (**b**) of SiC-based phase of composite SPSed at 1450 °C.

In contrast, the influence of the sintering temperature on the ISE of nanohardness is considerable for the Ti_3_SiC_2_ and SiC-based phases (Figure 10), because the phase composition and structure greatly depend on the diffusion process. An increase in the sintering temperature results in an increase in the Ti content in the SiC-based phase (former SiC particles), which is shown in Table 2. For this reason, the ISE is maximal for the high sintering temperature of 1450 °C (Figure 10c). However, the plastic deformation recourse of this phase remains minimal, and nanoindentation results in considerable distortion of the nanoindent (Figure 12a), as well as a small pile-up effect (Figure 12b). One can observe that crack initiation during loading (Figure 12a) results in the appearance of specific areas on the load-displacement nanoindentation diagrams (Figure 9b), leading to errors in calculating true hardness *H*_0_ and characteristic depth h* (Equation (4)).

The adequacy of the deformation behavior of the Ti_3_SiC_2_ MAX phase to SGP theory can be determined based on SGP equations (Equations (3) and (5)) and the experimentally defined parameters *H*_0_ and h* by calculating the densities of the GND and SS dislocations. Because *ρ_G_* = 4*γ*/*bD* [28], it is possible to state that the hardness square (Equation (3)) is inversely related to indent size *D*. Here *γ* is the average shear strain. The density of the SS dislocations may be determined based on Equation (5) and the resulting GND and SS dislocation dependence on the indentation depth for the Ti_3_SiC_2_ MAX phase will be defined in ongoing work.

## 4. Conclusions

This work presents the synthesis of Ti_3_SiC_2_ MAX phase-based composites using a Ti/SiC HEBMed and SPSed powder. The Ti_3_SiC_2_ MAX phase along with the TiC+Ti_5_Si_3_ phase were generated during SPS in the temperature range of 1300–1450 °C. The effects of SHS were found. It was shown that a higher sintering temperature of up to 1450 °C led to an increase in the Ti_3_SiC_2_ grain size. The effects of the Ti/SiC powder initial structure controlled by the HEBM process and the phase reactions during SPS on the mechanical properties of the composites were studied. The interdiffusion behavior of the atoms in the phases and at the interface of the phases significantly affected the mechanical properties of the composites. It was found that a thin TiC+Ti_5_Si_3_ layer was formed in situ on the SiC-Ti matrix interface via fast interdiffusion during the SPS process, which acts as a barrier hindering the diffusion of Si atoms from SiC to the Ti matrix. Diffusion of the Ti atoms into the large SiC particles in the applied sintering temperature range was found as well. The ISE was studied to define the deformation behavior of the SPSed Ti-SiC composite phases. It was determined that the ISE on the nanoscale is due to a change in the density of geometrically necessary dislocations in the Ti_3_SiC_2_ MAX phase, which is in agreement with SGP theory. The other TiC+Ti_5_Si_3_ and SiC phases do not exhibit deformation behavior based on SGP theory and have a low fracture toughness. Improvement of the Ti-SiC structure and properties is possible by optimizing the HEBM and SPS parameters to make a more uniform and controlled reaction sintering process.

## Figures and Tables

**Table 1:** Agglomerate size distribution of Ti/SiC HEBM powder.

D_10_, μm	D_50_, μm	D_90_, μm
17.6	42.2	82.2

**Table 2:** SEM-EDS analysis results of composites SPSed at 1350, 1400 and 1450 °C.

Phase Type	Sintering Temperature, °C	Phase Color	Area Number	Element Content, at%		
				Ti	Si	C
SiC particles	1350	Dark	1.1	07.63	51.82	40.55
		Gray	1.2	23.12	38.30	38.58
	1400	Dark	1.1	12.05	52.13	36.41
		Gray	1.2	34.09	29.50	33.45
	1450	Dark	1.1	09.99	56.18	29.56
		Gray	1.2	17.97	48.72	32.69
Ti matrix	1350	Gray	1.2	47.91	19.40	32.69
		Bright	1.3	43.47	18.84	37.70
	1400	Gray	1.2	47.79	25.77	26.45
		Bright	1.3	51.65	11.24	37.11
	1450	Gray	1.2	48.15	14.99	31.49
		Bright	1.3	47.68	39.32	13.00

**Table 3:** Effective density, hardness and fracture toughness of composites SPSed at 1350, 1400 and 1450 °C.

Sintering temperature, °C	Effective density, g/cm^3^	Hardness (HV_0.05_), GPa	Hardness (HV_0.5_), GPa	Hardness (HV_2_), GPa	Fracture toughness (K_1c_), MPa⋅m^1/2^
1350	4.33 ± 0.02	12.71 ± 0.39	11.54 ± 0.85	10.57 ± 0.7	n/a
1400	4.36 ± 0.01	14.06 ± 1.79	12.49 ± 1.85	11.55 ± 0.77	5.42 ± 0.47
1450	4.42 ± 0.01	16.34 ± 2.38	13.08 ± 0.96	12.83 ± 0.95	6.06 ± 0.02

**Table 4:** Nix and Gao equation fitting parameters of composites SPSed at 1350, 1400 and 1450 °C.

Test Type	Composite Phase	SPS Temperature, °C	Nix and Gao Equation Fitting Parameters		
			H_0_, GPa	h*, μm	R^2^
Nanohardness	Ti_3_SiC_2_	1350	48.5	0.110	0.99
		1400	41.8	0.069	0.96
		1450	51.8	0.103	0.87
	TiC+Ti_5_Si_3_	1350	25.4	0.069	0.81
		1400	29.5	0.108	0.86
		1450	19.8	0.086	0.74
	SiC	1350	56.2	0.032	0.97
		1400	53.0	0.072	0.93
		1450	69.2	0.191	0.97
Microhardness		1350	10.4	0.37	0.91
		1400	11.2	0.40	0.96
		1450	12.5	0.51	0.99

## Nomenclature

*λ*	Constant of Taylor equation (0.5)
*b*	Burgers vector
*D*	HEBMed powder agglomerate, μm
*F*	Indentation load, mN
*h*	Indentation depth, nm
*H*	Hardness parameter, GPa
*HV*_30_	Vickers hardness measured under the load of 294.2 N
*K*_1_ *_c_*	Fracture toughness, MPa⋅m^1/2^
*l*	Total length of cracks, μm
*μ*	Shear modulus
*ρ*	Dislocation density
*σ*	Normal stress
*T*	Temperature, °C
*τ*	Shear strength
*Θ*	Angle between the side surface of the pyramidal indenter and the base plane
